# Safety of perioperative period in robot-assisted atrial septal defect repair under hyperkalemic arrest

**DOI:** 10.1186/s40981-021-00436-w

**Published:** 2021-05-01

**Authors:** Kazuto Miyata, Tatsuya Tarui, Sayaka Shigematsu, Norihiko Ishikawa, Go Watanabe

**Affiliations:** 1 Department of Anesthesia, New Heart Watanabe Institute, Hamadayama 3-19-11, Suginami-ku, Tokyo, Japan; 2 Department of Cardiac Surgery, New Heart Watanabe Institute, Hamadayama 3-19-11, Suginami-ku, Tokyo, Japan

**Keywords:** Ultra-minimally invasive, Cardiac surgery, Robotic-assisted atrial septal defect repair, Hyperkalemic arrest

## Abstract

**Background:**

Various attempts have been made to meet patient desires, especially among younger and otherwise healthy individuals, for cosmetically satisfying incision with atrial septal defect (ASD) repair. One of procedures was a robotic-assisted totally endoscopic ASD repair via only two ports under hyperkalemic arrest without aortic cross-clamping. This study investigated perioperative management and safety for robotic-assisted total endoscopic ASD repair surgery under hyperkalemic arrest.

**Methods:**

We retrospectively reviewed perioperative management of thirty patients who underwent total endoscopic robot-assisted ASD repair under hyperkalemic arrest. All procedures were performed under general anesthesia using robotic-assisted total endoscopic for ASD repair via two or three ports under hyperkalemic arrest without aortic cross-clamping.

**Results:**

A total of 30 patients (mean age 45 ± 17 years, 8 male, 22 female) underwent successful ASD repair with the total endoscopic robotic-assisted procedures under hyperkalemic arrest.

Hyperkalemic arrest was achieved and maintained by intravenous administration of mean potassium dose of 91±32 mEq (1.4±0.6 mEq/kg) with the lowest bladder temperature was 31.9±1.4 °C during hyperkalemic arrest.

In all cases, serum potassium concentration was <5.0 mEq/L after weaning from cardiopulmonary bypass, although two cases who developed hyperkalemia >6 mEq/L after operation. At other time points, no patient exceeded 6 mEq/L of serum potassium concentration. At admission to the intensive care unit, mean serum creatine phosphokinase-MB level was 32±7mg/dL. There were no cases of arrhythmia or other cardiac complications during recovery.

**Conclusions:**

Perioperative management of robotic-assisted total endoscopic ASD repair under hyperkalemic arrest is safe and is not associated with fatal arrhythmia due to hyperkalemia.

## Background

In the field of cardiac surgery, robot-assisted techniques have been developed for coronary artery bypass, mitral valve repair, and for atrial septal defect (ASD) repair [1, 2]. For ASD repair, sub-mammary incisions or lateral thoracotomies are commonly used because of cosmetic reasons. We are performing robot-assisted totally endoscopic ASD repair (TER-ASD repair) via only two ports under hyperkalemic cardiac arrest without aortic cross-clamping [3]. Although this method has several advantages including no risk of aorta and left atrial appendage injury due to aortic cross-clamp as well as excellent cosmetic results with the reduced number of ports, there are only few reports evaluating the safety of surgery under hyperkalemic arrest [4]. Moreover, possible complications such as changes in perioperative potassium concentration, including fatal hyperkalemia, insufficient myocardial protection remains unsolved. The aim of this study is to investigate the safety of TER-ASD repair under hyperkalemic arrest from the points of potassium concentration and myocardial protection.

## Methods

### Patients

This is a retrospective data view of a single institute. After obtaining approval from The New Heart Watanabe Institute ethics committee (6 April 2020 and No. 2020-1), the authors examined the medical records of all patients received TER-ASD repair under hyperkalemic cardiac arrest from June 14, 2014, to February 9, 2018. Exclusion criteria were patients < 20 years, with the American Society of Anesthesiologist Physical Status (ASA-PS) ≥ 3, reduced cardiac function (ejection fraction <50%), histories of coronary artery, peripheral artery and lung diseases, renal dysfunction eGFR<60 ml/min/1.73 m^2^ and previous cardiac surgery.

### Anesthesia

General anesthesia was induced with midazolam 0.1 mg/kg, remifentanil 0.3−0.5 μg/kg/min, and rocuronium 0.9 mg/kg, followed by insertion of a left-sided, double lumen endotracheal tube. A normal endotracheal tube was used with a bronchial blocker in one patient in whom a double lumen endotracheal tube could not be intubated because of a small trachea. Anesthesia was maintained with sevoflurane (1−2%) and remifentanil (0.3−0.5 μg/kg/min) except the period of cardiopulmonary bypass (CPB), during which propofol (3−4 mg/kg/h) and remifentanil (0.2−0.3 μg/kg/min) were used. The lungs were ventilated with a pressure control mode during both one-lung and bilateral lung ventilation. Respiratory rate and peak plateau pressure (≤ 20 mmHg) were adjusted to maintain end-tidal carbon dioxide tension (ETco2) value below 45 mmHg. Bilateral lung ventilation was attempted if SpO2 decreased < 90%.

A transesophageal echocardiography (TEE) probe was inserted. A central venous catheter was placed in the left internal jugular vein and a 14−16 Fr venous cannula was inserted through the right internal jugular vein as drainage for the superior vena cava. The femoral arteries were usually cannulated with a 16-22 Fr arterial cannula; if the diameters of the arteries were narrow, both arteries were cannulated with a 12 Fr cannula. A 22−28 Fr venous cannula was inserted via the femoral vein and inferior vena cava, with its tip located in the right atrium.

### Surgical procedures

Two or three ports at the level of the fourth intercostal space in the left semi-lateral position were created. After establishing CPB, hypothermia was induced until bladder temperature decreases to 30-32 °C and ventricular fibrillation occurs, followed by infusion of potassium 1 mEq/kg to induce cardiac arrest. Additional potassium was injected if required for inducing cardiac arrest. Aortic cross-clamps were not placed.

ASD was directly closed using polytetra-fluoroethylene (Gore-Tex suture, W.L. Gore & Associates, Inc., Flagstaff, AZ, USA) after the right atrium was opened. De-airing was performed using left lung inflation before knotting the ASD defect. Serum potassium was filtered and removed out of the CPB using a hemodialyzer (PES-210Ex, NIPRO Inc., Osaka, Japan) after ASD was closed. Defibrillation (150 J) was used for ventricular fibrillation after ASD closure. The patient was weaned off the CPB after rewarming to 36.0 °C and serum potassium concentration < 5 mEq/L, followed by confirming absence of a residual ASD by TEE, administration of protamine, and removal of the cannulas in the right internal jugular vein and femoral vessels. In addition, we routinely administer loop diuretics at a dose of 20 mg at CPB weaning. If potassium levels exceed 5 mEq/L after CPB weaning, calcium gluconate and sodium bicarbonate (1 mEq/kg) are administered. If potassium levels remain above 5 mEq/L, a glucose-insulin solution (glucose 5 g/insulin 1U) is administered. The patient was then transferred to the intensive care unit (ICU) after changing the double-lumen endobronchial tube to a single-lumen tube. We measured potassium concentration using blood gas analyzer (GEM 3500 premier, Instrumentation Laboratory Inc., Massachusetts, USA)

### Outcome measurement

Postoperative cardiac function was evaluated by creatine kinase MB (CK-MB) at ICU admission and 1 day after operation. At 5 days after operation, we evaluated cardiac function and residual shunt of ASD using transthoracic echocardiography. Data are expressed as mean ± standard deviation.

## Results

**Fig. 1:**
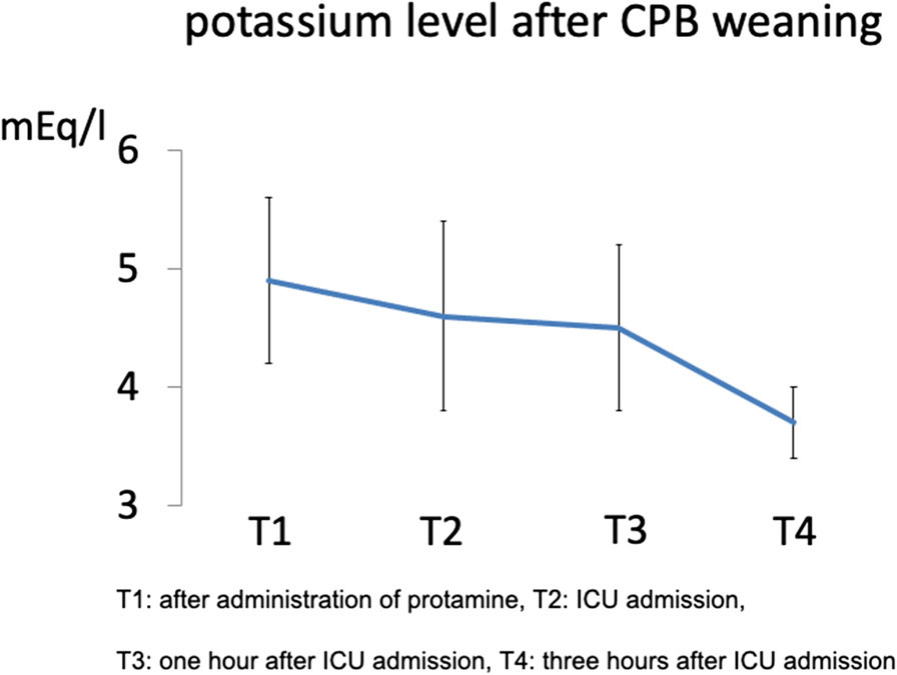
Potassium level after CPB weaning. T1, after administration of protamine; T2, ICU admission; T3, 1 h after ICU admission; T4, 3 h after ICU admission. The potassium level at T1 was 4.9±0.7 mEq/L, including two cases with levels exceeding 6 mEq/L. After ICU admission, the potassium level gradually decreased. Three hours after ICU admission, the potassium level was 3.7±0.3 mEq/L

Patients’ demographic data are shown in Table 1. Mean duration of surgery and anesthesia were 131 ± 32 and 243 ± 41 min, respectively; duration of cardiac arrest and CPB was 10 ± 4 and 56 ± 18 min, respectively. Robot-assisted surgery completed successfully in all patients without changing the surgical procedure such as median sternotomy and blood transfusion. There were no cases who developed SpO2 < 90% or ETco2 > 45 mmHg during one-lung ventilation. Cardiac arrest was induced by potassium 1 mEq/kg or > 1 mEq/kg in 8 or 22 cases, respectively. The mean dose of potassium injected during hyperkalemic arrest was 91 ± 32 mEq (1.4 ± 0.6 mEq/kg), with the maximum dose of 170 mEq (2.8 mEq/kg). The lowest bladder temperature was 31.9 ± 1.4 °C during cardiac arrest. The maximum potassium concentration exceeded the measurable level, i.e., 10 mEq/L, using continuous blood gas analysis (CDI 500 systems, TERUMO Inc. Tokyo, Japan) during CPB. Perioperative potassium concentration is shown in Fig. 1.

**Table 1:** Patients’ characteristics

Age (years)	45±17
Height (cm)	158±21
Body weight (kg)	60±12
Gender (Male/Female)	8/22
ASA classification 1/2	25/5
Serum creatinine (mg/dl)	0.67±0.16
AST (U/L)	20±7
ALT (U/L)	22±21
T-Bil (mg/dl)	0.8±0.5
EF (%)	67±7
Qp/Qs (%)	2.4±0.9
(Mean ±SD)

After ASD closure, thirteen patients (43%) experienced ventricular fibrillation, and 12 of these patients needed a single defibrillation to return from ventricular fibrillation to sinus rhythm, only one case needed the second single defibrillation to return to sinus rhythm. The rest of cases (57%) returned to spontaneous circulation naturally. After weaning from the CPB, there were no cases of residual ASD shunt and biventricular cardiac dysfunction. All cases were easily weaned from CPB under administration of dopamine 3 μg/kg/min.

Serum potassium level after CPB was > 6.0 mEq/L in two cases; > 5.5 and ≤ 6.0 mEq/L in four cases, although the mean potassium level was < 5.0 mEq/L after weaning off the CPB. It was < 6 mEq/L in all patients thereafter (Fig. 1). There were no cases of arrhythmia or bradycardia due to the hyperkalemia during recovery. Mean serum CK-MB was 32 ± 7 mg/dL and 13 ± 9 mg/dl on admission to the ICU and 1 day after surgery, respectively which returned to normal level in all cases. Transthoracic echocardiography 5 days after surgery revealed no residual ASD shunt and normal cardiac dysfunction with ejection fraction (EF) of 66 ± 4%. Duration from ICU admission to extubation was 5.0 ± 3.9 h. No patient required reintubation. There were no neurological complications, such as brain infarction and hemorrhage and no deaths.

## Discussion

In our case series, TER-ASD repair under hyperkalemic arrest was performed with no episodes of fatal hyperkalemia and arrhythmia or organ dysfunction during the postoperative period. This procedure was performed safely with good clinical results and excellent cosmetic outcomes.

Recently, robotic technology has progressed to provide cardiac surgeons with assistance that improves precision and accuracy. Moreover, as robotic-assisted cardiac surgery is ultra-minimally invasive, cosmetic concerns in ASD patients, especially those who are young and female, are resolved [3].

In terms of anesthetic management, robotic-assisted cardiac surgery has several concerns. One important concern is respiratory care during general anesthesia. Utilization of thoracoscopic ports requires the initiation of one-lung ventilation for adequate visualization of the cardiac structures. Moreover, insufflation of the left hemithorax with carbon dioxide is performed during robotic cardiac surgical procedures for adequate exposure of the heart and great vessels. In these condition, patients may experience arterial oxygen desaturation and hypercapnia because of one-lung ventilation and left artificial pneumothorax due to carbon dioxide. As hypoxemia and hypercapnia induce increased pulmonary artery vasoconstriction, right ventricular dysfunction may be caused by volumetric and pressure overload in patients with ASD. However, despite these concerns, in our series, there were no cases of desaturation as SPO2 below 90% and hypercarbia as ETCO2 above 50 mmHg, and no cases of right ventricular dysfunction.

There are several approaches to achieve myocardial protection without aortic clamping and cardioplegia, such as deep hypothermic cardiac arrest and hyperkalemic arrest [4–6]. Hyperkalemic cardiac arrest has been reported in reoperation for aortic valve replacement in a patient with a previous left internal thoracic artery to left arterial descending coronary artery bypass graft [4]. Compared to hypothermic cardiac arrest, hyperkalemia cardiac arrest is associated with decreased myocardial adenosine triphosphate levels [7]. In our series, serum CK-MB at ICU admission was slightly increased compared with the normal level. In all cases, 1 day after operation, serum CK-MB was decreased within normal levels which indicated that our hyperkalemia cardiac arrest technique provided efficient myocardial protection.

In our method, potassium was infused for cardiac arrest after inducing hypothermia. Hypokalemia caused by influx of extracellular potassium to intracellular compartments is frequently observed during hypothermia after cardiac arrest [8, 9]. In turn, intracellular potassium is moved to the extracellular compartment during rewarming, suggesting that hyperkalemia is caused by administered potassium as well as by transport from the intracellular compartment and its effective removal is crucially important.

To prevent postoperative fatal arrhythmia due to hyperkalemia, the most important procedure is to remove excessive serum potassium during CPB. In our series, the methods of reducing serum potassium levels included a dialyzer instead of a hemoconcentrator during CPB. The surface area of the dialyzer is 2.5 m^2^ compared with that of the hemoconcentrator, which is 1.2 m^2^. Serum potassium levels can be lowered more quickly using the dialyzer. However, compared with hyperkalemic arrest time, CPB time was much longer, due to the longer time required to reduce the potassium level below 5 mEq/L.

If potassium levels exceed 5 mEq/L after CPB weaning, calcium gluconate and sodium bicarbonate (1 mEq/kg) are administered. Indeed, two patients showed potassium concentration > 6.0 mEq/L and required calcium gluconate and sodium bicarbonate. If potassium levels remain above 5 mEq/L, a glucose-insulin solution (glucose 5 g/insulin 1U) is administered. Although calcium gluconate stabilizes the cardiac cell membrane against undesirable depolarization and sodium bicarbonate and glucose-insulin solution induce potassium shift back into the intracellular component, these effects are transient and the potassium cannot be removed in vivo. Thus, we routinely administer loop diuretics at a dose of 20 mg at CPB weaning. In our series, urine output has been sufficient after CPB weaning and there were no cases with hyperkalemia exceeding 6 mEq/L from ICU admission onward.

This study has some limitations. The patients were carefully selected to meet specific criteria. Patients did not have reduced cardiac function, coronary artery disease, peripheral artery disease, lung disease, renal dysfunction, or previous cardiac surgery. Another limitation is that this study was a retrospective analysis at a single institution. It would be difficult to generalize the results of this study to the general patient population.

## Conclusions

In conclusion, perioperative management of TER-ASD repair under hypokalemic arrest is safe and is not associated with fatal arrhythmia due to hyperkalemia.

## Data Availability

Not applicable

## Abbreviations

ASD: Atrial septal defect
TER-ASD repair: Totally endoscopic robot-assisted ASD repair
CPB: Cardiopulmonary bypass
ETCO2: End-tidal CO2
ICU: Intensive care unit
CPK-MB: Creatine kinase-MB
